# Community engagement in public health: a bibliometric mapping of global research

**DOI:** 10.1186/s13690-021-00525-3

**Published:** 2021-01-12

**Authors:** Ming Yuan, Han Lin, Hengqin Wu, Mingchuan Yu, Juan Tu, Yong Lü

**Affiliations:** 1 School of Civil Engineering, Zhengzhou University of Aeronautics, Zhengzhou, China; 2 Jiangsu Key Laboratory of Public Project Audit, School of Information Engineering, Nanjing Audit University, Nanjing, China; 3 College of Civil and Transportation Engineering, Shenzhen University, Shenzhen, China; 4 School of Finance and Business, Shanghai Normal University, Shanghai, China; 5 The Institute of Acoustics, School of Physics, Nanjing University, Nanjing, China; 6 College of Computer and Information Engineering, Hohai University, Nanjing, China

**Keywords:** Public health, Community engagement, Bibliometric, Visualization

## Abstract

**Background:**

Community engagement (CE) has been regarded as a critical element of successful health programs to achieve “the health for all” goals. Numerous studies have shown that it plays a significant role in reducing inequalities, improving social justice, enhancing benefits, and sharing responsibility towards public health. Despite this, the extant literature of community engagement in public health (CEPH) has topic-focused boundaries and is scattered across disciplinary. Large-scale studies are needed to systematically identify current status, hotspots, knowledge structure, dynamic trends, and future developments in this field.

**Methods:**

The bibliometric techniques were applied in the analysis of publications on CEPH in Web of Science Core Collection from Thomson Reuters. One thousand one hundred two papers out of 70.8 million publications over the period of 1980 to 2020 and their 15,116 references were retrieved as the sample set. First, basic characteristics of publications, including distributions of geography, journals and categories, productive authors and frequently cited articles, etc. were obtained. Then, four bibliometric methods, i.e. social network analysis, co-citation analysis, co-occurrence clustering, and burst detection, were further conducted to sketch the contours of the structure and evolution of CEPH.

**Results:**

Between Jan 1, 1980, and Apr 25, 2020, CEPH has attracted a sharp increase in interest all over the world. Total 117 countries or regions have participated in the field of CEPH and the contributions are geographically and institutionally distinct. The United States is the key region performing such research, which accounts for more than half of the total number of publications. Developing countries, such as South Africa, India, Brazil and China also contributed a lot. The advancements of CEPH are marked by historically momentous public health events and evolved from macroscopic strategies to mesoscopic and microscopic actions. Based on keyword clustering and co-citation clustering, we propose a 4O (i.e. orientation, object, operation, and outcome) framework of CEPH to facilitate a better understanding of the current global achievements and an elaborate structuring of developments in the future.

**Conclusion:**

This study draws an outline of the global review on the contemporary and cross-disciplinary research of CEPH which might present an opportunity to take stock and understand the march of knowledge as well as the logical venation underlying research activities which are fundamental to inform policy making.

## Introduction

The lethal rampage of COVID-19 makes a thorough revelation of the fragment and fragility in the current global public health system [1–3]. To meet the big challenges, the traditional paradigm of public health is confronting urgent calls to be reshaped and strengthened from the exclusively vertical, top-down and curative scheme to an inclusive, whole-of-society, and people-centered one [4–8]. Community engagement (CE), involving communities in decision-making and in the planning, design, governance and delivery of services [9, 10], has been regarded as effectively responding means to offer the fundamental support for public health by reducing isolation and improving social capital) [11]. To put the “public” into public health, CE first came to prominence in the public health arena since the 1978 Alma Ata Declaration [12]. It reflected a revolutionary thinking that health is not only an outcome of biomedical interventions, but also a result of social determinants [13–15]. In the following period, the concerns on CE proliferated [16–18]. After the SARS crisis, 196 countries across the globe elaborate an international legal instrument, the International Health Regulations, laid stress on working with communities in response to the international spread of disease in 2005 [19]. Later, both WHO report of the Commission on the Social Determinants of Health [20] and World Report on Primary Health Care [21] highlighted the important role of CE in empowerment and local capacity building again [22]. In 2016, WHO Framework on Integrated People-Centred Health Services re-emphasized CE as one of its key strategies [23]. In the age of COVID-19, pandemic control increasingly relied on CE which could ensure thoughtful applications of diverse measures with respect for specific contexts and rights [6, 8].

In accordance with the idea that there is no public health without community supporting [24], many counties have embraced CE to address public health problems [5, 25–28]. China’s barefoot doctors, India’s rural health centers, Nigeria’s community health extension workers, Malawi’s health surveillance assistants, Ghana’s Navrongo Experiment, and Basic Development Needs Programme in Eastern Mediterranean Region are all typical examples [13, 28, 29]. Evidence has shown that CE plays a significant role in reducing inequalities, improving social justice, enhancing benefits, and sharing responsibility towards public health [9, 25, 30, 31]. Ironically, despite the recognition that CE is a critical element of successful health programs to achieve “the health for all” goals [22], engaging communities has still been somehow under-represented or “lost” in promoting public health with grave consequences [13, 32]. For instance, sluggish response to early disease events often impaired the rapid detection and headstream control while communities are the first to know unusual things happened [11]. Lack of data tracking and limited community ownership constrained the coverage and left the poor behind [13]. The extreme manifestation of community fear could even lead to the killings of health workers [7]. Achievement of high and equitable coverage public health requires long-term decision making, elaborate designing, better local training, and supportive supervising. Burgeoning the power of community, revitalizing CE, and learning from the decades of experience is crucial to reconstruct public health systems in all countries, both developed and developing.

In lined with the prominent emergence of community engagement practice in public health sector, a wide range of academic publications related to this relevant topic are contributed from worldwide institutions and organizations, and with diversification into various disciplines. Since these publications have topic-focused boundaries and are scattered across disciplinary, a robust synthesis of the research is needed to pull the literature together in a coherent way and an agreed terminology for future development. However, to the best of our knowledge, the extant literature fails to provide a big and fine-grained picture of academic research on community engagement in public health (CEPH) which could systematically describe current status, hotspots, knowledge structure, dynamic trends, and future developments in this field. As a quantitative approach, the bibliometric analysis could provide an in-depth and comprehensive understanding of specific research areas involved based on a large-scale publication [33, 34]. Therefore, aiming to fulfill this gap, we adopted a longitudinal bibliometric analysis to draw an outline of the global review on the contemporary and cross-disciplinary research of community engagement and public health. The technology-based review presents an opportunity to take stock and understand the march of knowledge as well as the logical venation underlying research activities which are fundamental to inform policy making.

## Methods

### Overview

The bibliometric analysis involved a large-scale assessment of more than 70.8 million articles in Web of Science Core Collection from Thomson Reuters. It aimed to conduct a timely and comprehensive literature review on CEPH between 1973 and 2020, and to identify significant opportunities for future research. Although the earliest article on the subject of our research appeared in 1973, this document appeared before the year of Alma Ata declaration, 1978, and there was no in-depth study of the subject in the following 7 years. Therefore, we exclude this document and choose the first document after the Alma Ata declaration appeared in 1980 for thematic analysis. Beyond the boundaries of traditional reviews which often based on the authors’ own knowledge, opinions, and experience, the bibliometric analysis employs quantitative methods to probe diverse aspects of scientific communication as well as the knowledge structure and evolution trajectory of published documents [33].

Bibliometrics plays an important role in showing the current status of research in a certain field or discipline, identifying important journals and scholars in the field, delineating the knowledge structure of the discipline field and tracking the dynamic evolution of the development of the field. Compared with the traditional review method, bibliometrics has obvious advantages in extending content and time span, being free from professional knowledge, and excavating objective information [35].

**Fig. 1:**
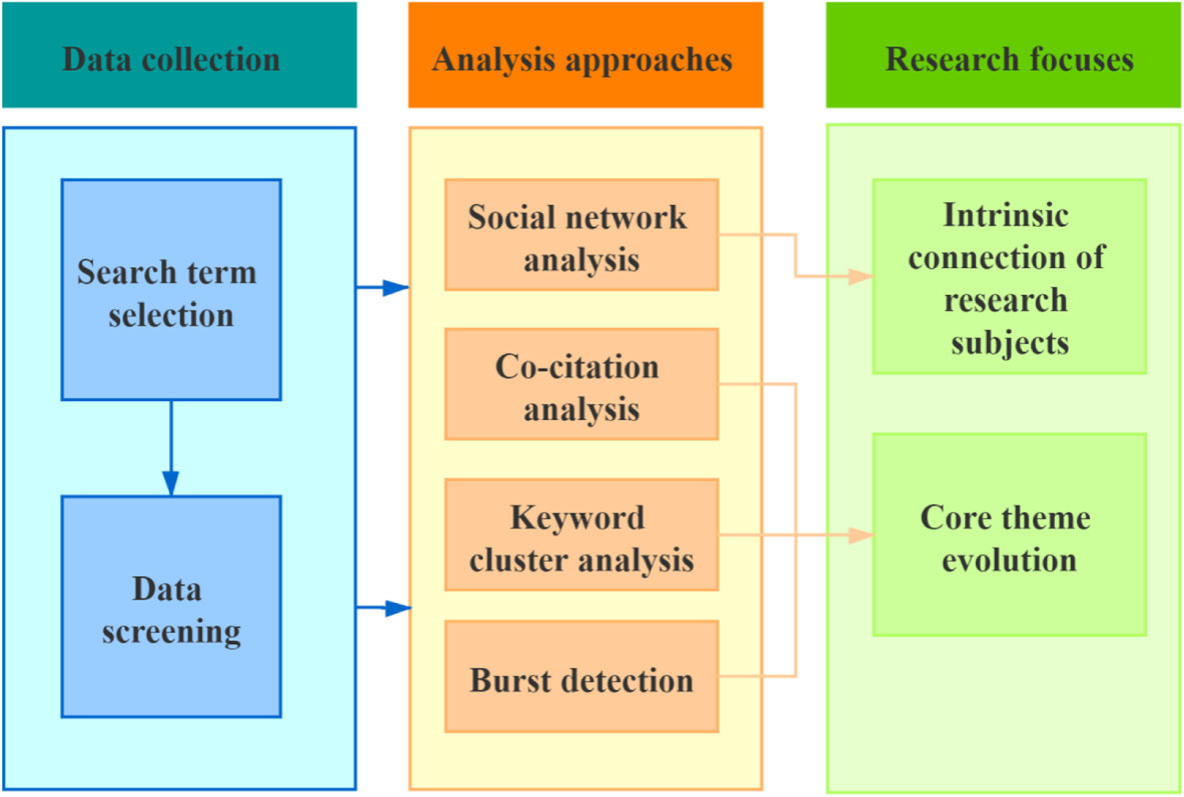
The research framework of this study

As a result, more fine-grained and objective results could be provided than typical author-scoped reviews. In the current study, we followed the three steps for conducting bibliometric analysis (see Fig. 1). While the sample is consisted of publicly available data from academic documents, ethics review, and approval was not required at this time.

### Data collection

Given the complexity of the concept, it is not surprising that there are various expressions defining “*community engagement*” [25, 32]. For example, community involvement and community participation both connote manifestations of community engagement [28]. Finding relevant publications with the avoidance of drawing a narrow search boundary raises particular challenges [27, 33]. Two practical strategies were employed to identify relevant studies. First, expert interviews were conducted to help the search term identification. Second, Web of Science Core Collection was selected as the original database which fully contains the mainstream international academic journals over a long period and offers detailed information, especially the references, of publications. Finally, the searching strategy resulted in the following scheme: ((“community engagement” OR “community participation” OR “community involvement” OR “community consultation”) AND (“public health”)). Between January 1, 1980 and April 25, 2020 (data acquisition time), 1102 publications and their 15,116 references were retrieved as the sample set.

### Analysis approaches

Four emerging bibliometric methods were applied in the current study: social network analysis, co-citation analysis, co-occurrence clustering, and burst detection, which formed the structure and evolution of CEPH.

#### Social network analysis

Rooted in graph theory, social network analysis facilitates the visualization of knowledge network structures [36]. We can learn about the relational traits of publications (e.g. the associations among authors, research institutions, journals, and countries) in the Create Citation Report section in the Web of Science Core Collection, which enhances the understanding of studies’ hidden information and prominence in an academic domain by calculating particular parameters (e.g. degree, betweenness, and closeness centrality).

#### Co-citation analysis

Co-citation refers to a relationship of a pair of documents that are simultaneously cited by other articles [37]. It is generally used to present the similarity of content between the pair documents. The number of the co-citations signals the influences of the cited work due to the underlying rationale that the bibliographic references form the theoretical and empirical foundation of the scientific study citing them [38]. The co-citation patterns in scientific literature could help to excavate pivotal publications in terms of citation popularity, upsurge, and network.

We use computer assembly language to set the existing co-citation relationship to “1” and the non-co-citation relationship to “0”, construct a co-citation co-occurrence matrix, and import the matrix into the “Gephi” software to visualize the co-citation relationship. Gephi provides a modular program to cluster co-citation to better see the internal structure of the subject.

#### Co-occurrence analysis

Co-occurrence refers to a phenomenon in which the information described by the characteristic items (such as title, author, keywords, institution, etc.) of the literature co-occurs. Keywords reflect a highly condensed topic and content of a publication. The joint occurrence of keywords within a set of publications indicates their close relationships of themes [39]. Treating co-occurrence like co-citation, thus we cluster similar studies to assess the strength of the linkage between keywords. In the consequential clusters, top keywords that have a high frequency of occurrence clearly sketch the contours of the core themes and contents in a specific research field [40]. Co-citation clustering could also shed light on the knowledge structure, relationship network and evolution process of specific fields, and then the focused research topics of the target literature [41].

#### Burst detection

Burst detection is used to analyze a set of keywords or publications to recognize intellectual turning points based on the changes of specific characteristics which exhibit high intensity over a short period of time [42]. CiteSpace provides the burst detection function module to detect large changes in the number of keywords or citations in a certain period of time, to find the decline or rise of a certain topic. A keyword or co-citation with high burst score demonstrates the fast-growing interests among researchers. The introduction of burst analysis could describe the eruption, evolution, and decay of research topics and themes as well as the gravity shifting of research hotspots [43].

## Results

### Research trend

#### Overall trend

**Fig. 2:**
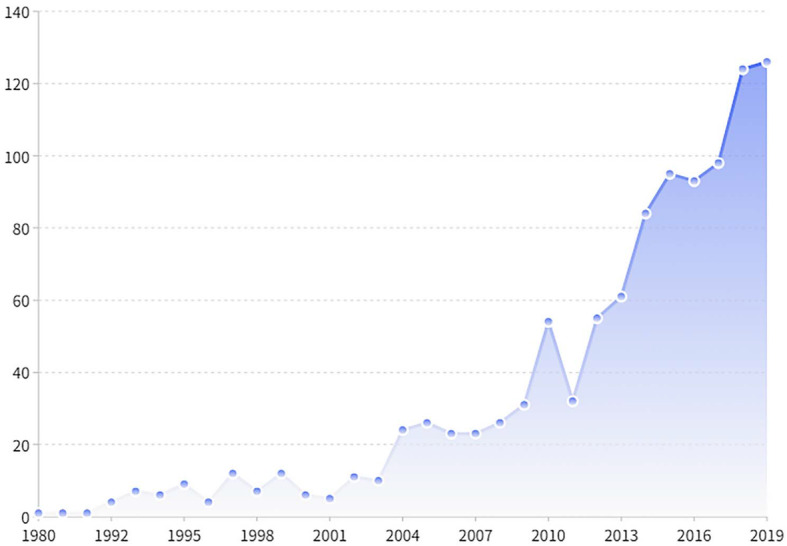
The annual number of CEPH publications

Since 1980, the number of publications of CEPH has been steadily increasing year-on-year, exhibiting the increasing interest of the academic community in this field, as shown in Fig. 2. However, there is a significant difference between the two period from 1980 to 2003, and 2004 to 2020. The first period grew smoothly only accounting for 8.96% of the total publications while the second period accounting for 91.04%. By and large, exponentially growing CEPH has been one of the most influential and dynamic fields of health policy research due to *cause celebres* in the last two decades.

##### Country distribution

**Fig. 3:**
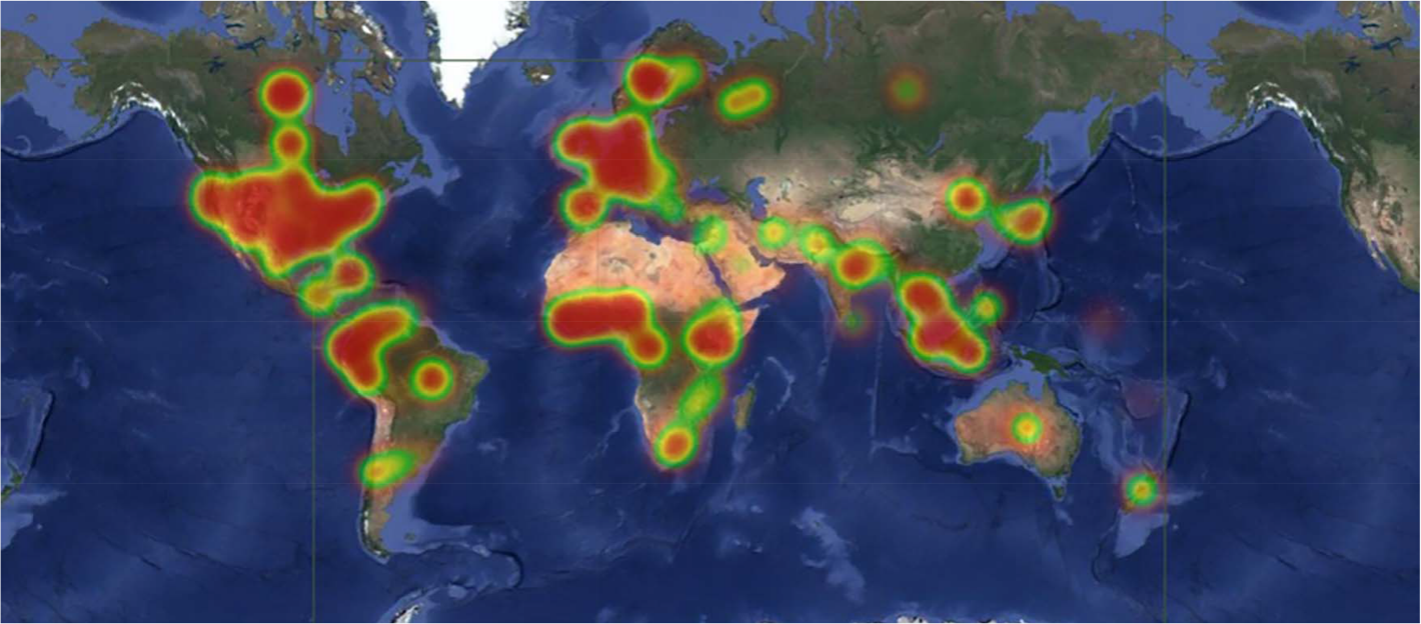
Geographical distributions of CEPH publications

Figure 3 draws the geographical location of organization that have contributed to CEPH and Table 1 lists numbers of publications and H-index of the most productive 10 countries. The color depth in the figure is proportional to the number of publications, which means the denser the color, the greater the number of articles. It can be seen that the regions with densest red dots are North America and Europe.

**Table 1:** The top 10 countries of CEPH

Rank	Country	Numbers of publications	H Index
1	USA	576	46
2	ENGLAND	156	25
3	CANADA	105	24
4	AUSTRALIA	90	18
5	SOUTH AFRICA	41	12
6	SWITZERLAND	41	18
7	INDIA	33	11
8	BRAZIL	30	8
9	NETHERLANDS	29	10
10	CHINA	26	12

Total 117 countries or regions were involved in CEPH, both developed and developing countries participate. The top 10 countries in the number of publications are mainly distributed in the Americas, Europe, Africa and Asia, which means that more emphasis is placed on issues related to community engagement in public health in these countries compared to other countries. As the country with the highest number of articles published, the United States publishes 51.11% of the world’s total publications in CEPH, which has established its leading position in this field. The United States has the highest H index of 46, and far higher than other countries.

As the developing countries in the top 10 countries, South Africa, India, Brazil and China occupy a large number of seats. The reason for this distribution seems obvious: these countries have been plagued by epidemic diseases, and the residents have great health and survival pressure, which forces the countries to look for new ways to improve the current health care pressure.

##### Institutional analysis

Table 2 ranks the top ten productive institutions including specific information. *Johns Hopkins University* is the institution that publishes the most articles, with a total of 70 articles, accounting for 6.35%. Three of the top five institutions belong to the United States. It is worth mentioning that, among the top five institutions, *Johns Hopkins University* is the agency responsible for the statistics of the new coronary pneumonia in the United States. The fifth-ranked *World Health Organization* promotes the prevention and treatment of epidemics and endemic diseases and plays a guiding role in improving public health.

**Table 2:** The top 10 productive institutions of CEPH

Rank	Institution	Type	Numbers of publications	Proportion(%)
1	JOHNS HOPKINS UNIVERSITY	University	70	6.35
2	UNIVERSITY OF CALIFORNIA SYSTEM	University	65	5.90
3	UNIVERSITY OF LONDON	University	53	4.81
4	CENTERS FOR DISEASE CONTROL PREVENTION USA	Government agency	44	3.99
5	WORLD HEALTH ORGANIZATION	United Nations agency	41	3.72
6	UNIVERSITY OF NORTH CAROLINA	University	34	3.9
7	LONDON SCHOOL OF HYGIENE TROPICAL MEDICINE	Research institute	32	2.90
8	UNIVERSITY OF WASHINGTON	University	31	2.91
9	UNIVERSITY OF WASHINGTON SEATTLE	University	29	2.63
10	NATIONAL INSTITUTES OF HEALTH NIH USA	Research institute	27	2.45

##### Author analysis

Table 3 lists the top ten most productive authors in the CEPH, as well as their H index, total citations, and average citations per item. Among them, England and USA both have three authors, and one from Kenya, China, Australia, Cuba, meaning most productivity authors come from developed countries. The author with the largest total number of articles is South J. from England. A total of 8 articles have been published with an H index of 4 and a total of 65 citations. The highest number of total citations is *Cargo M.*, who comes from Australia, with an average of 130.25 citations per article.

**Table 3:** The top 10 productive authors of CEPH

Rank	Author	Numbers of publications	Country	H index	Total Citations	Average citations per item
1	South J.	8	England	4	65	8.13
2	Schoch-Spana M.	7	USA	3	41	5.86
3	Wells K.B.	6	USA	5	167	27.83
4	Kawachi I.	5	USA	5	293	58.60
5	Molyneux S.	5	Kenya	5	88	17.60
6	Thomas J.	5	England	4	102	20.40
7	Tucker J.D.	5	China, USA	5	47	9.40
8	Brunton G.	4	England	4	100	25.00
9	Cargo M.	4	Australia	4	521	130.25
10	Castro M.	4	Cuba	3	62	15.50

Notably, two authors *Wells K.B.* and *Cargo M.* are found to lead to citation bursts, which shows that these two scholars are field pioneers and play an important role in leading research frontiers and topics.

##### Journal analysis

Global scholars published articles in 483 different international journals. As can be seen from the Table 4, listing the top 10 journals by volume. These journals belong to 103 different categories. *Public, Environmental & Occupational Health* ranks first in research domain, followed by *Environmental Sciences & Ecology*, *Health Care Sciences & Services*, *Biomedical Social Sciences*, etc. A total of 150 articles were published in the top 10 journals, accounting for 13.61% of the total.

**Table 4:** The top 10 productive journals of CEPH

Rank	Source Titles	Numbers of publications	Quartile in Category	Impact Factor	Research Domain	H index
1	BMC PUBLIC HEALTH	41	Q2	2.567	Public, Environmental & Occupational Health	15
2	JOURNAL OF PUBLIC HEALTH MANAGEMENT AND PRACTICE	29	Q3	1.42	Public, Environmental & Occupational Health	7
3	INTERNATIONAL JOURNAL OF ENVIRONMENTAL RESEARCH AND PUBLIC HEALTH	25	Q2	2.468	Environmental Sciences & Ecology; Public, Environmental & Occupational Health	6
4	PUBLIC HEALTH	25	Q2	1.696	Public, Environmental & Occupational Health	11
5	AMERICAN JOURNAL OF PUBLIC HEALTH	23	Q1	5.381	Public, Environmental & Occupational Health	15
6	HEALTH PROMOTION INTERNATIONAL	17	Q2	1.913	Health Care Sciences & Services; Public, Environmental & Occupational Health	8
7	SOCIAL SCIENCE MEDICINE	17	Q1	3.087	Public, Environmental & Occupational Health; Biomedical Social Sciences	12
8	CRITICAL PUBLIC HEALTH	16	Q1	2.742	Public, Environmental & Occupational Health; Biomedical Social Sciences	6
9	PROGRESS IN COMMUNITY HEALTH PARTNERSHIPS RESEARCH EDUCATION AND ACTION	15	Q4	0.64	Public, Environmental & Occupational Health	3
10	AMERICAN JOURNAL OF PREVENTIVE MEDICINE	12	Q1	4.435	Public, Environmental & Occupational Health; General & Internal Medicine	8

##### Highly cited publications analysis

Table 5 shows the top 10 highly cited publications. The most cited article is *Community-Based Participatory Research Contributions to Intervention Research: The Intersection of Science and Practice to Improve Health Equity*, published by *Univ New Mexico* in the journal *American Journal of Public Health* in 2010 [44], it has been cited 650 times with an average of 65 times a year. In this article, authors identified the obstacles and challenges faced by Community-based participatory research (CBPR) as well as the proposes to improve the imbalance of rights CBPR. Rank second and third papers are from the same journal, *Annual Review of Public Health*, which has made a great contribution to CEPH. The former solves the problem of minority groups such as African Americans and blacks access to health equity. The latter reviews the public health literature comprehensively, constructs a comprehensive practice framework including developing and maintaining participatory research partnerships, designing and implementing participatory research efforts, and evaluating the intermediate, and long-term outcomes.

**Table 5:** The top 10 frequency cited publications of CEPH

Rank	Author	Year	Title	Journal	Total Citations	Average citations per item
1	Wallerstein, N. et al.	2010	Community-Based Participatory Research Contributions to Intervention Research: The Intersection of Science and Practice to Improve Health Equity	AMERICAN JOURNAL OF PUBLIC HEALTH	650	65.00
2	Yancey, A.K. et al.	2006	Effective recruitment and retention of minority research participants	ANNUAL REVIEW OF PUBLIC HEALTH	610	43.57
3	Cargo, M. et al.	2008	The value and challenges of participatory research: Strengthening its practice	ANNUAL REVIEW OF PUBLIC HEALTH	409	34.08
4	Catalani, C. et al.	2010	Photovoice: A Review of the Literature in Health and Public Health	HEALTH EDUCATION & BEHAVIOR	399	39.90
5	Israel, B.A. et al.	2005	Community-based participatory research: Lessons learned from the Centers for Children’s Environmental Health and Disease Prevention Research	ENVIRONMENTAL HEALTH PERSPECTIVES	273	18.20
6	Utzinger, J. et al.	2005	Conquering schistosomiasis in China: the long march	ACTA TROPICA	250	16.67
7	Lasker, R.D. et al.	2003	Broadening participation in community problem solving: a multidisciplinary model to support collaborative practice and research	JOURNAL OF URBAN HEALTH-BULLETIN OF THE NEW YORK ACADEMY OF MEDICINE	234	13.76
8	Campbell, C. et al.	2000	Health, community and development: Towards a social psychology of participation	JOURNAL OF COMMUNITY & APPLIED SOCIAL PSYCHOLOGY	205	10.25
9	Harris, S.B. et al.	1997	The prevalence of NIDDM and associated risk factors in native Canadians	DIABETES CARE	199	8.65
10	Raviglione, M.C. et al.	2002	Evolution of WHO policies for tuberculosis control, 1948–2001	LANCET	154	8.56

### Burst detection

#### Burst literature

**Fig. 4:**
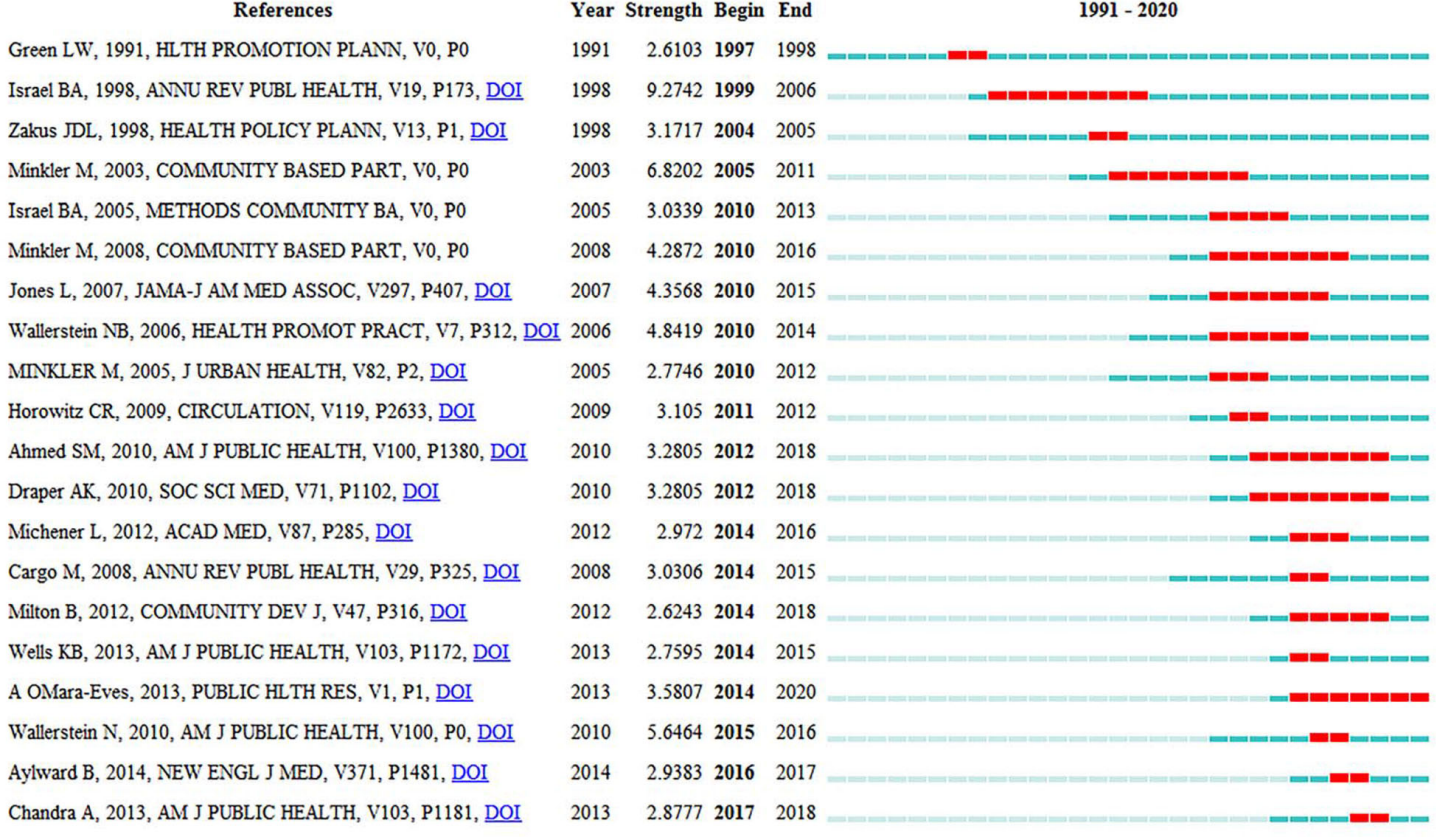
Top 20 references with the strongest citation bursts of CEPH publications

Figure 4 lists the 20 publications with the highest “leading trend” in the obtained data. The sudden increase in citations of a publication means that it has received special attention at the very stage, and may make distinguish contributions to the academic field.

Major literature of the top 20 publications focused Community-Based Participatory Research (CBPR). For example, Wallerstein & Duran [45] “*Using community-based participatory research to address health disparities*” (Ranked 8th), Wallerstein & Duran [44] “*Community-based participatory research contributions to intervention research: The intersection of science and practice to improve health equity*” (Ranked 18th), Horowitz et al. [46] “*Community-based participatory research from the margin to the mainstream are researchers prepared?*” (Ranked 10th), and Minkler [47] “*Community-based research partnerships: Challenges and opportunities*” (Ranked 9th) all touched this topic through various perspectives. These seminal publications began to increase citations after 2010, which indicates that CBPR has produced a series of innovative thinking and deeply penetrated considerable factors that affect health and disease, such as building partnerships with researchers and local community workers, sharing resources, and exchanging ideas and expertise with each other.

Draper et al. [32] “*Chasing the dragon: Developing indicators for the assessment of community participation in health programmes*” (Ranked 12th) proposed an assessment framework by capturing multiple ways of community health participation and assesses the role of community participation in improving health. The article came in sight in 2012 and shed light on how to evaluate the effectiveness of community participation in improving public health in a comprehensive way.

Michener et al. [48] “*Aligning the goals of community-engaged research: Why and how academic health centers can successfully engage with communities to improve health*” (Ranked 14th) demonstrated that academic health centers (AHCs) should better interact with community participation research in the future in accordance with the following 5 steps: defining community and identifying partners, learning the etiquette of community-engaged, building a sustainable network of community-engaged researchers, recognizing that community-engaged research will require the development of new methodologies, and improving translation and dissemination plans. Attention to this article began in 2014, suggesting that, from then on, AHCs are beginning to tell in the field of public health [48].

Aylward et al. [49] “*Ebola virus disease in west Africa — The first 9 months of the epidemic and forward projections*” (Ranked 19th) retrospected the evolution process of the first 9 months of the Ebola virus epidemic and predicted future development. It highlighted the role of community health participation in epidemic outbreak control in the developing areas.

Wells et al. [50] “*Applying community engagement to disaster planning: Developing the vision and design for the Los Angeles County Community Disaster Resilience Initiative*” (Ranked 16th) and Chandra [51] et al. “*Getting actionable about community resilience: The Los Angeles County Community Disaster Resilience Project*” (Ranked 20th) gave prominence to Community Resilience (CR) which is in accordance with the progress of other disciplines [50, 51]. Since 2014, a growing number of studies have boosted the discussions and advanced new principles, frameworks, policies, and methods to build community resilience.

#### Burst keywords

**Fig. 5:**
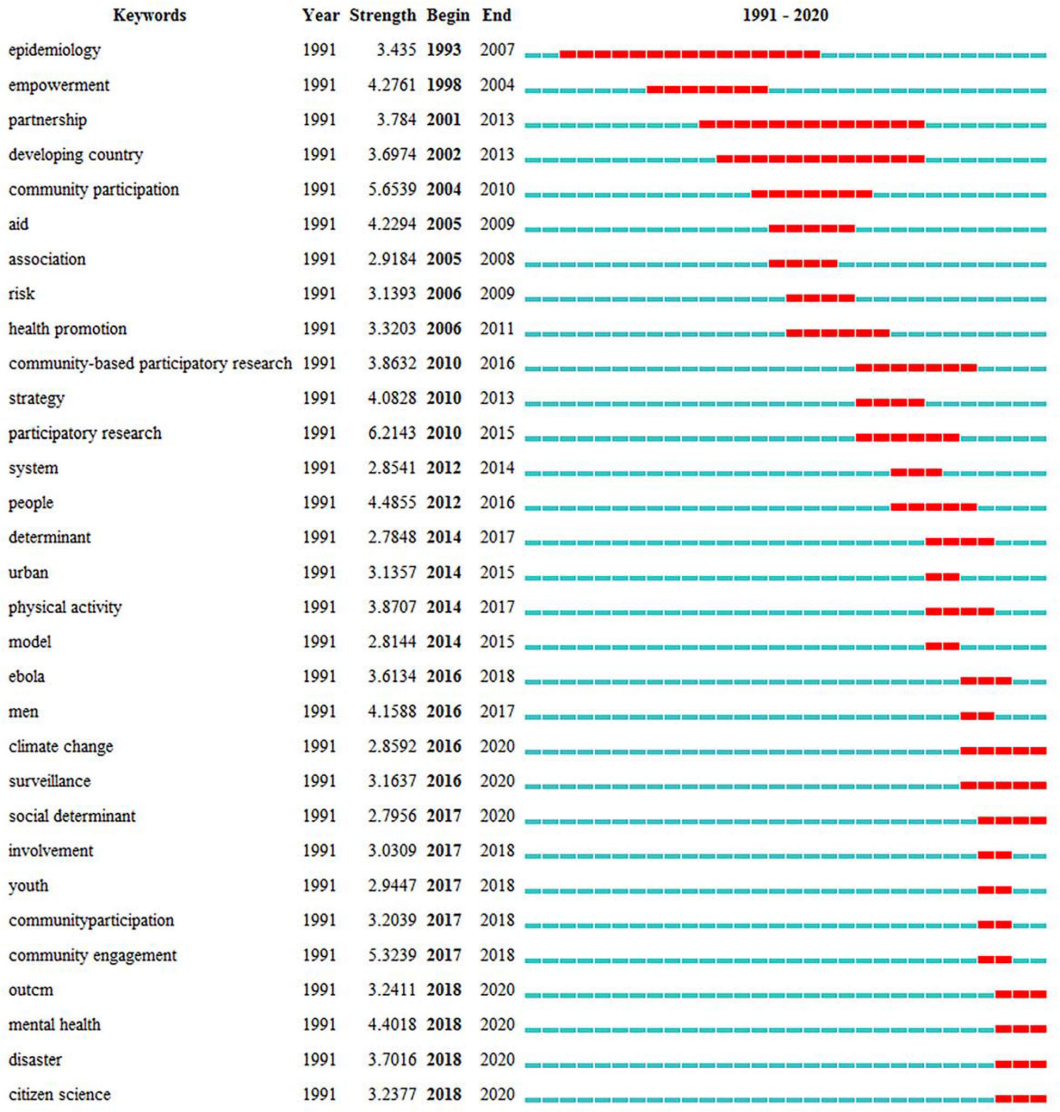
Top 31 keywords with the strongest citation bursts of CEPH publications

Figure 5 shows the strongest intensity of the top 31 keywords in the CEPH and their dynamic evolution from 1991 to 2020. It is obvious that before 2010, the hotspots of CEPH were mainly associated with relatively abstract topics, such as *aid, health promotion, association,* and *empowerment*. After 2014, the interests in CEPH shift to more specific subjects, including *men, youth, climate change, social determinant, disaster, ebola,* and *mental health*. The narrowing down transition exhibited the consistent changes between burst literature and keywords and formed distinct characteristics of the CEPH development.

Altogether, the analysis of burst keywords and burst literature exhibits the clear evolution progress of CEPH. First, the feature of overall research changes from macro framework design to the medium-micro content investigation. In the early stage, CEPH studies mainly focused on exploring the ultimate purpose, deducing or conceiving the feasible path, and designing the theoretical framework. In the wake of CEPH advancements, scholars have turned their attention to deeper understanding and more specific contents, such as focusing on individual healthcare, targeted chronic diseases, and infection prevention and control (2014–2020).

Second, the evolution of CEPH research reflects vivid age-related features. For example, the outbreak of “Ebola” or “SARS” instantly fueled the academic debate and attracted audiences around the globe, so does the “COVID-19”. To meet these challenges, an international organization, Citizen Science Global Partnership, was founded to explore how citizens can help monitor progress towards the UN’s sustainable development and health equity goals. Then, citizen science in CEPH has begun to take shape and has been increasingly emphasized. Inevitably, “emergency events” with the imprint of the times marked the evolution of CEPH research in recent years.

### Keyword co-occurrence and co-citation clustering

**Fig. 6:**
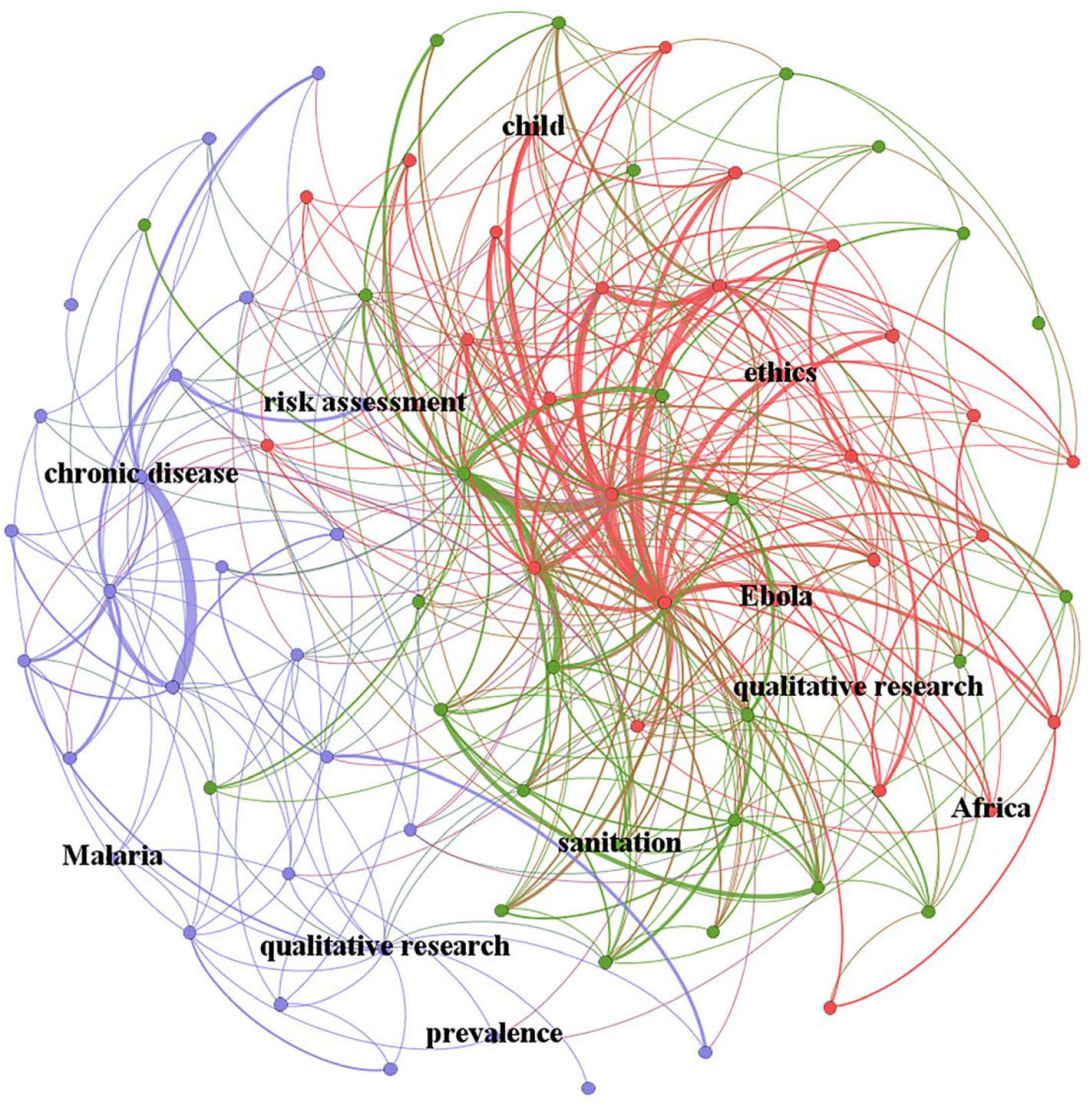
The keywords co-occurrence cluster analysis of CEPH publications

**Fig. 7:**
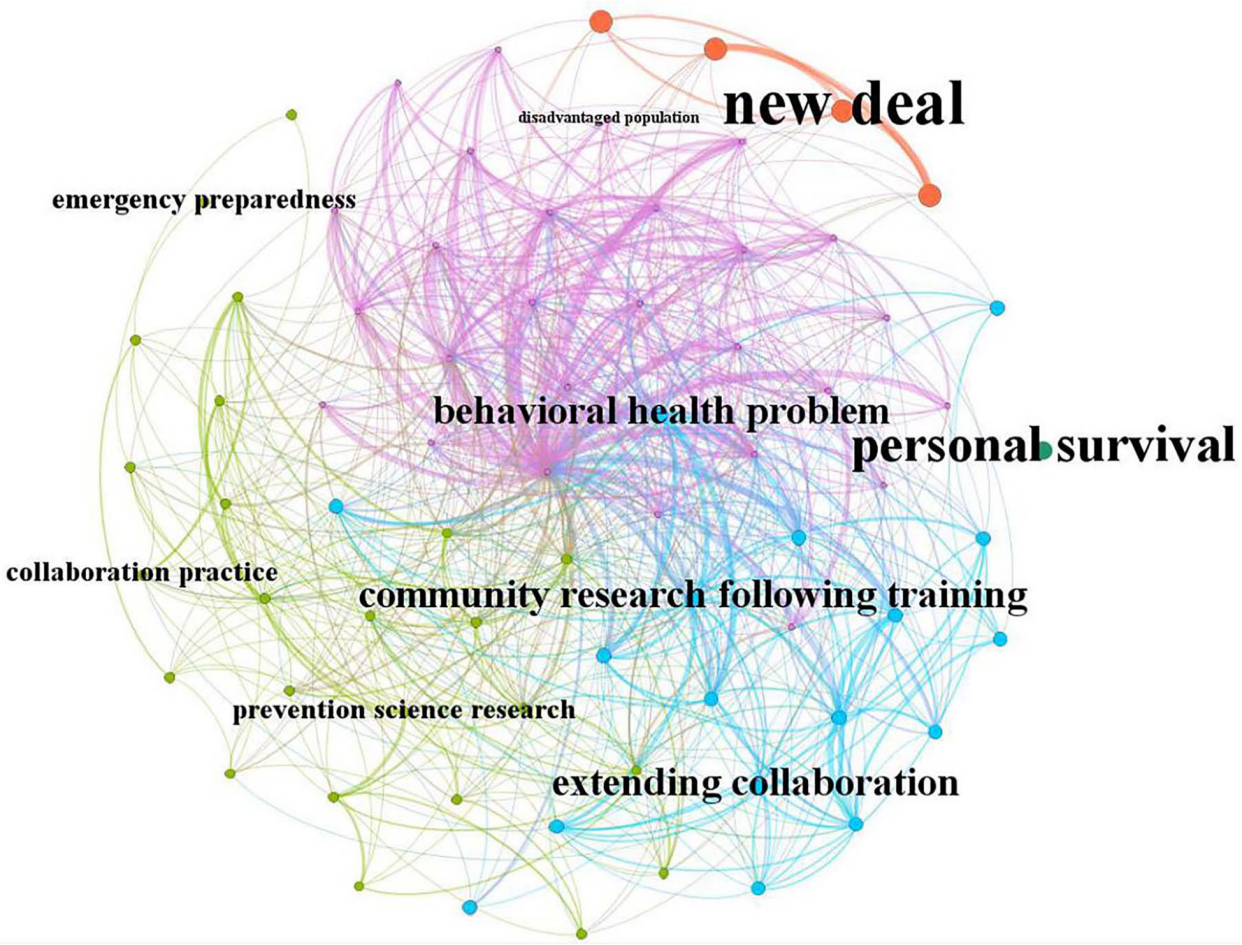
The co-citation cluster analysis of CEPH publications

**Fig. 8:**
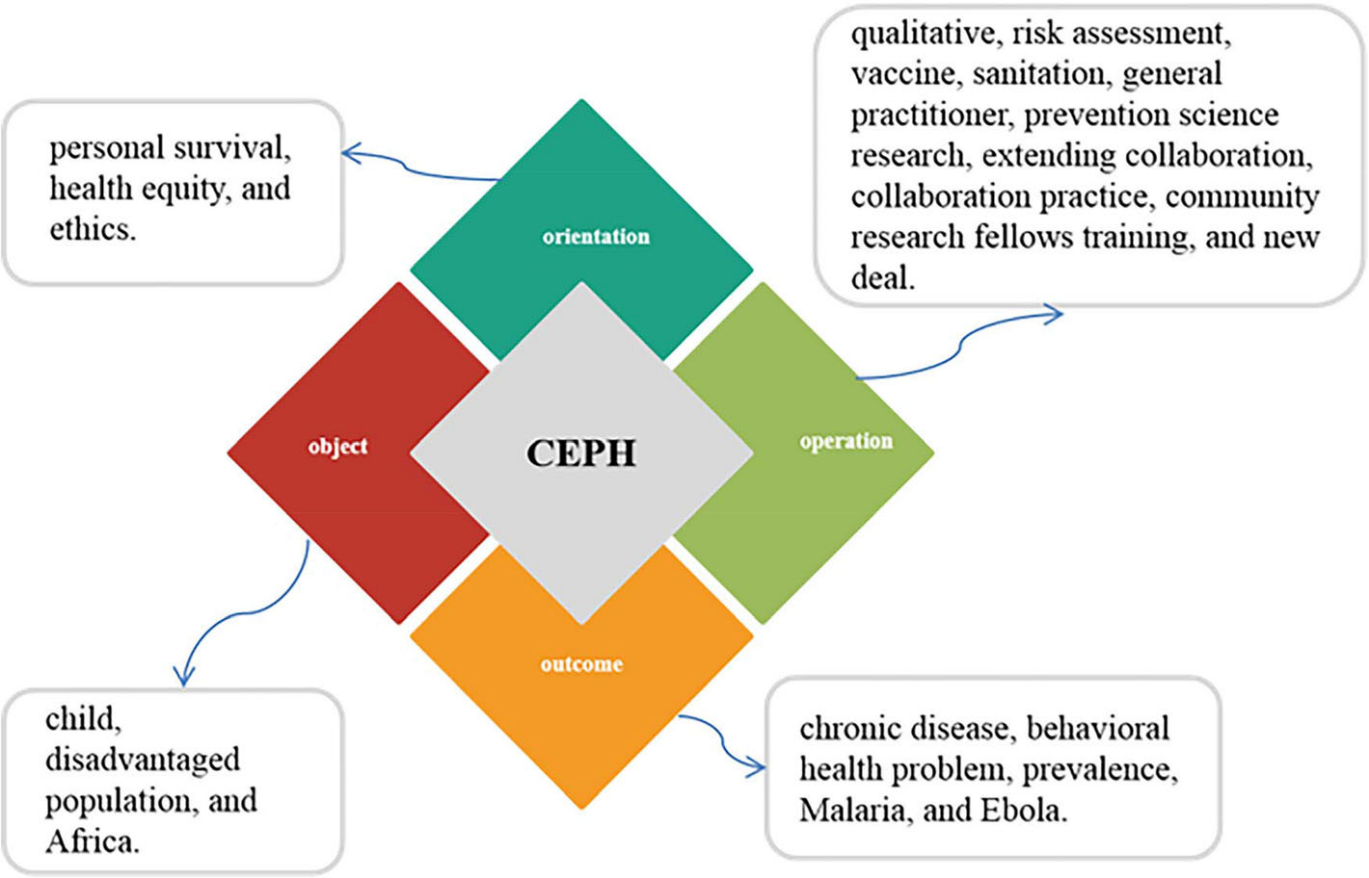
The 4O diagram for CEPH

The keyword co-occurrence and co-citation clustering maps (Figs. 6 and 7) were created to identify the core themes and the structure of related to the research about CEPH. According to the visual and statistical analysis, the clustering pattern emerged as the following four dimensions: *orientation*, *object*, *operation*, and *outcome* (See Fig. 8).

The “orientation” dimension delineates the particular interests, activities, or aims of the research about CEPH towards its practical significance or theoretical development. The current literature in this cluster mainly focused on topics related to *personal survival*, *health equity*, and *ethics*. The goal of public health is“the health for all”which consists of 3P - short for prevention of diseases, prolong life, and promotion health [52]. The underlying thinking of this ultimate aim reflect serious humanistic solicitude that fairness and morality are just as important to human beings as the individual survival. Communities, in which people live or work closely together and share the same risks [28, 32], have emerged as typically embodied organizations to meet the development of public health [13, 25, 26]. Efforts coordinated by communities could facilitate infectious disease control [53], personal hygiene education [54], medical service delivery [55], and environmental improvements [56], and ensure every community member’s basic health resources and equity [44, 57]. Engaging communities also involves spontaneous and autonomous activities complementary to institutional void or resource constraint, especially in developing countries. Nationwide community mobilization demonstrates deep commitments to collective action and enforce the aggressive disease containment which helps China get a jump on the COVID-19 pandemic [58]. Thus, community engagement towards personal survival, health equity, and ethics clearly annotate the spirit of public health.

The “object” dimension refers to a target or a particular group that community engagement is directed at within the context of public health. The major research in this cluster concentrated efforts on *child*, *disadvantaged population*, and *Africa*. To realize the pursuit of public health, the most fundamental and challenging problem is the vulnerable groups and regions. Children, woman, and disadvantaged people (e.g., people with low socioeconomic or be socially excluded) are the typical populations who need extraordinary concerns [13, 27]. Community engagement, relying on innovative health-care delivery embedded in the community, could timely respond to objective and expressed health demands and overall development by reducing alienation from society for the vulnerable members [59]. In Africa, the continent suffering from serious health issues, strengthening weak ties to community groups and institutions does facilitate obtaining information and resources and linking to opportunities. This significantly improves the underlying determinants of health, such as the environment, agriculture, education, and livelihoods, and then leads to better morbidity, mortality, and health inequalities [27]. Since the planet is all interconnected in terms of health and well-being, there is an urgent call for providing convenient health and other essential services, and protecting the most vulnerable among us based on community engagement, not only for children and women, but also for ethnic minority, indigenous, immigrant communities, and displaced people.

The “operation” dimension involves the actions or methods that affect the public health improvement through engaging communities. The relevant issues in this cluster included the keywords: *qualitative*, *risk assessment*, *vaccine*, *sanitation*, *general practitioner*, *prevention science research*, *extending collaboration*, *collaboration practice*, *community research fellows training*, and *new deal*. These keywords specify the main measures or methods of CEPH. That is, engaging communities currently emphasizes cooperation, focuses on prevention, and employs qualitative research methods. General practitioners are still the core suppliers of health service. Sanitation and vaccine are regarded as two cardinal strategies to offer basic living services for people and prevent the spread of the epidemic [60]. While collaboration is increasingly highlighted in recent years, substantial disruptions in operations have also been reported from the field. The traditional interventions confined to health facilities only offered faint glimmers without implementation within communities. Big challenges exists to build ability to accurately grasp the complex place-and-time-specific contexts inter-coupling with contemporary public health emergence [61]. How community engagement could be designed properly and implemented through effective ways is the guarantee to lead public health on track to reach the goal.

The “outcome” dimension reflects the results or effects of community engagement on public health. Significant studies were crowned with achievements in *chronic disease*, *behavioral health problem*, *prevalence*, *Malaria*, and *Ebola*. This dimension is the tangible performance evaluation of CEPH due to specific issues. Involving infectious, chronic and mental disease, public health has a broadening scope related to everyone. Engaging community for public health can be considered to be both conceptually distinct but also practically purposeful in its effects. It could not only fight against epidemics in a collaborative manner, but also alleviate psychological disorder (e.g. depression, PTSD) and build interpersonal trust. For chronic diseases, communities might be the gatekeeper, which is responsible for outpatient follow-up and medication guidance for their ill members, and actively conduct interventions to reduce the rate of injury, disability, and mortality of chronic diseases, then improve health status and quality of life [15]. Since results-based evaluation is an incentive assessment of a planned, ongoing, or completed intervention, the focuses of final outcome stemmed from CEPH would ensure its relevance, efficiency, effectiveness, impact, and sustainability. Despite of great progresses, large gaps still exist in the current practice. The lack of systemic design leads to a fragmented situation that various departments independently operate in their own ways. This discordance often brings about duplication of efforts, an exercise in futility, and even a backfire. Inverse care law furtherly distorts the allocation of resources and services resulting in the acute shortage of coverage in the regions that urgently need healthcare on the ground [11, 62]. Formal and informal institutions are not unanimous proponents of the recognition that there is no public health without community supporting [24, 63]. Community engagement mechanism is not mature enough when citizens’ rights of participation and supervision are not guaranteed [64].

**Fig. 9:**
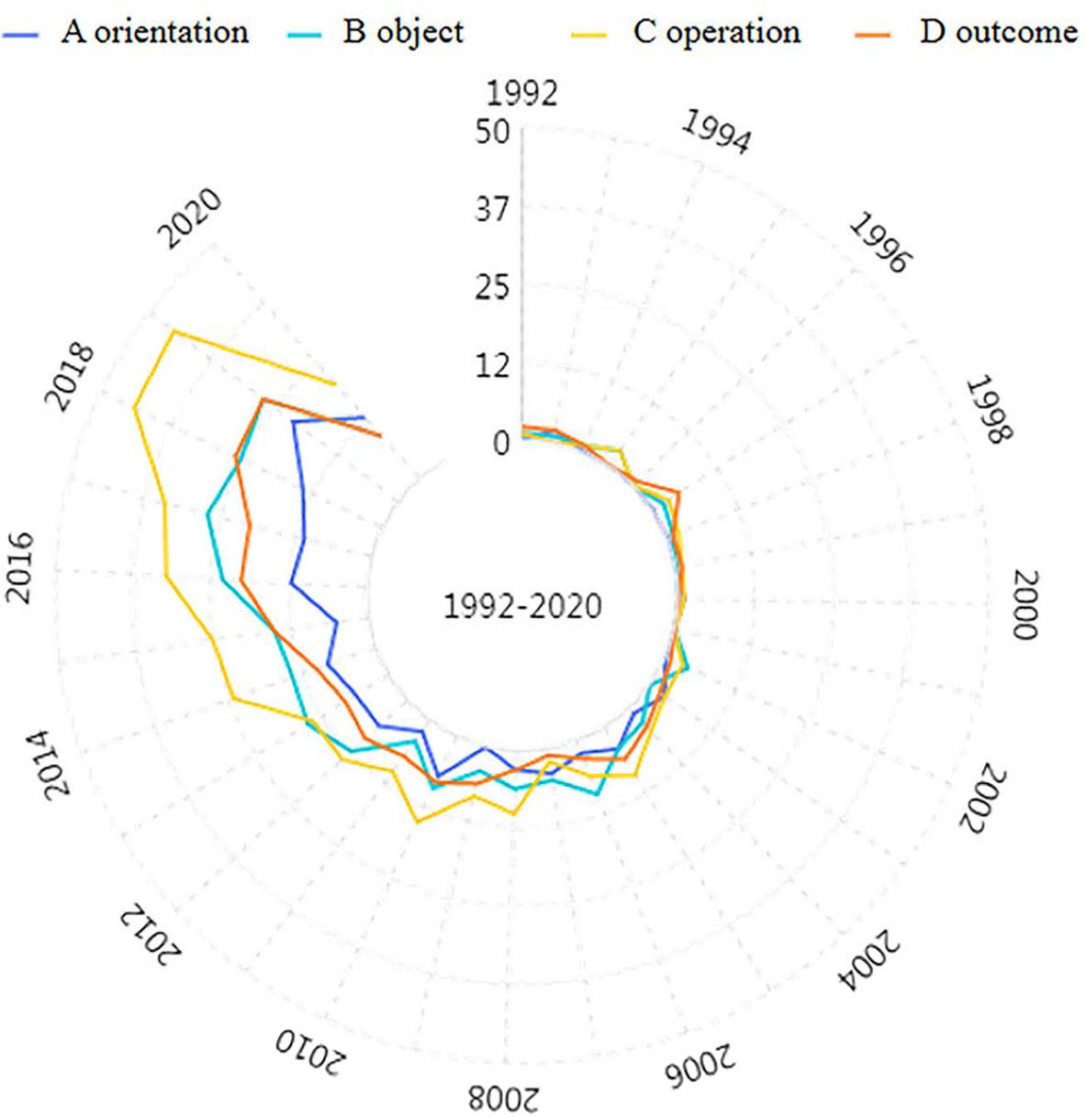
Timeline of publications of CEPH in the 4O dimensions

To show the longitudinal patterns related to the 4O dimensions, a quantitative visualization based on the annual number of publication belonged to each dimension is given in Fig. 9. The results are familiar with the findings of burst analysis. In the first half of the time, the distribution of 4O is uniform. As people began to focus on to the level of healthcare, scholars are encouraged to pay more attention to new techniques, methods or applications responding to the emerging public health problems. *Object* and *outcome* dimensions are evenly consistent. On the contrary, the *orientation* dimension has the least amount of published articles due to its abstract characteristics. The advancements in *orientation* would have a bearing on foundations of CEPH.

## Discussion

Historically, interventions and actions to promote public health are dependent on professionals with little or no input from the targeted people [27]. Urgent calls from practitioners, policymakers, and researchers have been to engage members of the communities [6, 25]. By enhancing the social and environmental determinants that underpin public health, engaging communities alleviates social, economic, and demographic inequalities, with the influence eventually felt by all populations [18, 28]. Academic progresses on CEPH provide theoretical and technical guidances on community engagement as well as independent assessments of its health effects. The evidence is clear that interventions by community engagement have serious implications for our health, wellbeing, and the evolution of organized society. Its direct effects result from proximal surveillance, instant response, accurate information, convenient services delivery, and improved intervention efficacy [11]. The impacts of community engagement will also be mediated through less direct pathways, including changing health behaviors, building trust, strengthening social capital, and reducing inequalities [10, 27]. Although many of these effects are already identified, the absence of elaborate community engagement strategy will potentially amplify existing global public health challenges. Typically, the lagging community engagement does not perfectly work for much of the world’s population in terms of providing an integrated, synergistic response to COVID-19. People, with limited cognition on community engagement, often only rely on assistance from community residents or communal general practitioners. Insufficient training in up-to-date healthcare knowledge constrains the prevention of complicated situations. The traditional interventions confined to health facilities only offered faint glimmers without implementation within communities. Thus, engaging local communities needs the highest priority and skilled social worker and community staff have to be better integrated in response teams since it would be inevitable that responding to public health issues in diverse environments will become more common. Equally essential, therefore, will be an improved understanding of challenging operational contexts among affected communities and external responders alike [11].

This quantitative synthesis identified trends in CEPH that can be considered when designing future policies. A comprehensive and ambitious scheme of community engagement could greatly transform the health of the world’s populations. Monitoring and ensuring this transition, from an opportunity to a reality, is the central duties of practitioners, policymakers, and researchers. There is indeed a long way to go before the ultimate goal of public health can be achieved. To highlight the role of community engagement, an inclusive, whole-of-society strategy and systemic people-centered approaches should be implemented in the coming future. The following recommendations, which are based on the results observed in the bibliometric analysis, might be helpful for researchers and practitioners.

First, policy frameworks should be built for engagement to happen in a coordinated way. Governance of institutions, leadership, collaborations, and interventions are all imperative to integrate expertise, resources, and capacity through national and regional public health institutions. Gaining full and concerted support from governments, funding agencies, and health professionals, community members are inclined to achieve good outcomes in health through proactive community participation. Governments and the global health community need to learn from the past experiences of Ebola and Zika viruses and the recent outbreaks of COVID-19, another slow response without local communities will result in an irreversible and unacceptable cost to human health. Engagement requires to start before an outbreak—ensuring that patients, their families and their communities are in the coverage is essential for the successful public health. There is no public health without the support of the community [11].

Second, a mismatch of demand and supply ought to be ended in terms of fair access to care. Health systems are weakest where the needs are greatest [9]. The outbreaks of public health events have brought heavy burdens to both less developed regions countries and the vulnerable populations [6, 8]. There is solid evidence that community engagement interventions have a positive impact on a range of health and psychosocial outcomes, across various conditions [27]. Engaging a community in action to address the provision of healthcare services could help ensure the principal of “equal treatment for equal need.” By improving information flows that are effective for the planning, design, delivery or governance of health services, community engagement would change the availability of medical care to vary with the right need for it in the population served. Taking into account the diversification, personalization, and familiarization, community-based resource allocation tends to facilitate the elimination of health inequalities and inequities.

Third, novel formal or informal measures are supposed to be embraced to dealing with critical issues around ownership, empowerment, education, mobilization, and sustainability of health improvements. Prominent incentive- or monitoring-based initiatives to promote greater community involvement must ensure that information disclosure is transparent and that the community members’ voice is heard, including a direct reporting system, testimonial sessions, self-help health groups, and so on. For example, a new organizational model, community-based human services organizations or CBOs, could be boosted to build ability to grasp the complex place-and-time-specific contexts inter-coupling with contemporary epidemic emergence, and is essential for public health control. Meanwhile, social entrepreneurial measures or tools are expected to be adopted at community level for the successful prevention and response to epidemics. Self-help health groups are complementary social networks against the exhaustion of medical resources and miscommunication, mistrust, and fear stemmed from epidemics. Also, communities are encouraged to consolidate the neighborhood friendship, build the community consciousness of “same breath, common destiny”, and strengthen the relationship construction and emotional interaction based on a community health platform.

Fourth, novel assessments are expected to be developed to guide the future progress. Towards the goal of “everyone enjoys health care,” the new evaluation schemes should not only emphasize the final outcome, but also facilitate the whole process of dynamic control and adjustment by combining qualitative and quantitative methods. Concrete indicators need to be well tailored to the safe, affordable, convenient, and acceptable healthcare for all. Besides the infectious diseases, greater attention on chronic and mental diseases is needed in a collaborative manner. Communities could be responsible for outpatient follow-up and medication guidance, and then reduce the rate of injury, disability, and mortality of non-infectious diseases. To be a qualified health gatekeeper for all community members, a complete network for grassroots public emergency, community-level acute epidemiological investigation, and key disease surveillance is in desperate need for communities. It not only provides long-term dynamic information, including the timing and location of the frequent occurrence of diseases, screen high-risk groups, but also ensure the outcomes of the comprehensive public health strategies. In order to consolidate the positive results achieved by the existing community and provide better health services for the community residents, wider coverage and scope should also be expanded in the future. On the one hand, more investments are requisite for infrastructure construction, health equipment up upgradation, and medical care supply. On the one hand, advanced active interventions, such physiological counseling, healthy diet, and physical exercise, are expected to be vigorously introduced to the whole community.

## Conclusions

Over the past four decades, the development of CEPH has attracted a sharp increase in interest all over the world, especially since 1980. A good many of scholars and institutions have made significant contributions to the scientific advances in CEPH over the period 1980–2020 that have been brought to light. By a bibliometric analysis on a sample of 1102 relevant publications and their 15,116 references, this study tries to draw the outline of the global review on the latest research and that of newly emerging topics of CEPH. The evolution trajectory, based on the quantity of literature, geographical and periodical distribution, national comprehensive strength, journal distribution, productive authors and institutions as well as category, citations, keyword co-occurrence, and co-citation analysis, is drawn to present research hotspots, valuable ideas, and developing tendency of CEPH in a global context.

Briefly, CEPH has entered an exponential development stage and caught the world’s attention. Total 117 countries or regions have contributed to this field. Most of the research is carried out in North American and European countries, and African countries occasionally get involved. The advancements of CEPH are marked by historically momentous public health events and evolved from macroscopic strategies to mesoscopic and microscopic actions. A four-dimension framework (orientation, object, operation, and outcome), due to the clustering pattern, is proposed to facilitate a better understanding of the current global achievements and an elaborate structuring of developments in the future.

## Data Availability

Data involved a large-scale assessment of more than 70.8 million articles in Web of Science Core Collection from Thomson Reuters, which aimed to conduct a timely and comprehensive literature search on community engagement in public health between 1983 and 2020.

## Abbreviations

CE: Community Engagement
CEPH: Community Engagement in Public Health
4O: orientation, object, operation, and outcome
COVID-19: Corona Virus Disease 2019
WHO: World Health Organization
